# The process of social death in patients with hip fracture

**DOI:** 10.1038/s41598-023-50115-2

**Published:** 2024-01-19

**Authors:** Golnar Ghane, Zahra Zare, Hooman Shahsavari, Shirin Ahmadnia, Babak Siavashi

**Affiliations:** 1 Department of Medical Surgical Nursing, School of Nursing and Midwifery, Tehran University of Medical Sciences, Tehran, Iran; 2 Department of Operating Room, School of Allied Medical Science, Tehran University of Medical Sciences, Tehran, Iran; 3 Department of Sociology, School of Social Sciences, University of Allameh Tabataba’i, Tehran, Iran; 4 Department of Orthopedics, School of Medicine, Tehran University of Medical Sciences, Tehran, Iran

**Keywords:** Psychology, Environmental social sciences, Diseases, Health care, Health occupations, Medical research

## Abstract

People with chronic disability and uncontrollable long-term complications following hip fracture have characterist.ics that may predispose them to social death. Continuous physical disability can have negative physical, psychological, and social consequences in these patients. To design care interventions for preventing and controlling social death, it is essential to identify the dimensions and characteristics of this process. Therefore, the present study aimed to explain the process of social death in hip fracture patients. In this study, which was conducted using a grounded theory approach, 20 patients were selected with maximum diversity and 9 professional and non-professional caregivers also through purposive sampling followed by theoretical sampling. Data were collected through semi-structured in-depth interviews, field notes, and observations. Data were analyzed using the approach proposed by Corbin and Strauss in stages including data analysis for concepts and their dimensions and characteristics, the context, process extraction, and integration of the categories. One core category and 16 main categories, which consisted of 55 subcategories and 212 primary concepts, were extracted. The results showed that the core process of social death in hip fracture is an intentional self-destruction for getting liberated from the conditions of the illness and the disrupted social life after the fracture, which ultimately leads to outcomes such as isolation-seeking and death ideations in these patients. The core category of liberating self-destruction reflects the close relationship between the context, process, and outcomes of social death. The process of social death is social, multidimensional, and complex. So far, no explanatory theory has been presented for this group of patients. Therefore, the results of this study can play an important role in designing helpful interventions for preventing, modifying, and changing the phenomenon of social death.

## Introduction

Hip fracture is one of the most common orthopedic injuries and a serious and complex condition reported as one of the top ten causes of morbidity, mortality, and increased healthcare costs in today’s world^1,2^. In addition to prolonged hospitalization and major surgeries, these patients also require repeated rehabilitation after discharge and long-term care to improve and recover their mobility^3,4^. The results of studies have shown that despite the various medical advances, the improvement and recovery of mobility after hip joint fracture has not changed dramatically over the past 25 years^5,6^, and the mortality rate is still high in individuals with hip fracture^7,8^.

From a thanatological perspective, there are several types of death, including biological or physical death, psychological death, and social death. It is, however, difficult to distinguish between the different types of death in patients, as they are closely tied to one another^9,10^. Social death is a condition that often occurs with social isolation and the loss of identity and social role among older adults and individuals with chronic disabilities that do not improve^9,11,12^.

From the perspective of social scientists, social death is a multi-dimensional concept that includes rejection (being ignored or excluded by others or oneself)^13^, social marginalization and loneliness, and loss of personality and a valuableness of life and is closely linked to changes in the individual’s identity and role in life^9,14^. The symptoms of social death are not only psychological but also physical. For example, rejection may even cause physical pain. In addition, these conditions may accelerate the individual’s mental, psychological, and physical death^15,16^. Social death becomes apparent when the person gets unable to participate socially and is considered dead while still alive. After reviewing extensive articles on social death, Králová argued that social death consists of three main elements: the loss of social identity, loss of social relationships, and deficiencies related to physical inefficiency^17^.

Social death has not been adequately addressed in patients with hip fracture, but individuals suffering from this type of fracture often spend their days waiting for death without sufficient attention from the society, family, and even healthcare providers^2,18^. In Iran, evidence suggests that individuals with hip joint fracture are discharged after surgery and initial treatment without any follow-up by the healthcare providers, community, and occasionally even their families^19,20^. As a result, many of these patients lack family and social support and also the necessary facilities to perform their personal activities independently, and these deficiencies can lead to higher rates of postoperative complications, poor living conditions, and even social death.

In the present study, the main hypothesis was based on the symbolic interaction theory. Thus, to understand the process of social death formation in individuals with hip joint fracture, one should consider the perspectives, beliefs, and experiences of these patients, their social position and interactions with others, their interpretation of their social status and interactions after hip joint fracture, and social and personal factors that may facilitate or impede the formation of social death, as well as any other problems and conditions emerging from social death. Given the above hypotheses, the process of social death is highly complex and cannot be explained in simple terms. Often, events are the product of multiple causes that interact in a complex and unpredictable way^21^. Therefore, a method seeking to understand experiences and explain situations can be helpful in discovering the process of formation of this phenomenon.

The concept of social death in patients is complex and multidimensional and has not yet been studied in the sociocultural context of Iran. Also, this concept has not been studied in individuals with hip fracture. Therefore, the various aspects of the phenomenon are unknown and further studies are needed for its clarification and in order to identify the interaction patterns of these patients so as to enable providing better and more thorough care to these patients. Conducting a grounded theory study on this subject thus seemed helpful.

## Methods

This qualitative study was conducted using the grounded theory approach by Corbin and Strauss^21^ from 2020 to 2022. The participants of this research were individuals with hip joint fracture who were or had been hospitalized in the orthopedic wards of Tehran University of Medical Sciences hospitals. The samples were selected through purposive followed by theoretical sampling with maximum diversity.

The study received approval from the Ethics Committee of Tehran University of Medical Science (TUMS) with the number (Number: IR.TUMS.FNM.REC.1398.217). The patients were fully informed about the purpose and methods of the study, and they were informed that they could withdraw from the study at any time. Informed consent was obtained from all participants, and they were assured that their answers would remain confidential. All the methods are in accordance with relevant guidelines and regulations.

The settings in which the samples were accessed consisted mainly of the inpatient and outpatient orthopedic wards of Tehran University of Medical Sciences hospitals. Additionally, for theoretical sampling and to discover the process of social death formation during the treatment period, the participants were visited at their home or nursing homes after their acute treatment phase.

The study included 29 participants, including 20 patients with hip fracture, two family caregivers, two non-family caregivers, two orthopedic ward nurses, one social worker, one physiotherapist, and one orthopedic resident. The researcher attempted to include individuals with diverse experience in social death formation to achieve maximum diversity and theoretical saturation. Table 1 presents the characteristics of the participants.

**Table 1:** Demographic characteristics of study participants.

Participant	Age	Gender	Marital Status	Education	Duration of treat	Socio-economic status
Patient	51	Female	Divorced	Master’s degree	1years	Good
Patient	59	Female	Widow	High school diplomas	45 days	Moderate
Patient	34	Male	Single	Bachelor's degree	35 days	Moderate
Patient	84	Male	Married	Bachelor's degree	14 days	Good
Patient	79	Female	Widow	Junior school	60 days	Moderate
Patient	43	Female	Married	Bachelor's degree	6 months	Moderate
Patient	48	Male	Married	Bachelor's degree	4 months	Moderate
Patient	26	Female	Single	Bachelor's degree	2 months	Good
Patient	55	Female	Married	Bachelor's degree	1 years and 6 months	Moderate
Patient	60	Male	Married	Junior school	6 months	Moderate
Patient	76	Male	Widow	Bachelor's degree	5 months	Moderate
Patient	70	Female	Married	High school diplomas	1 years and 4 months	Moderate
Patient	45	Male	Married	High school diplomas	1 years	Good
Patient	65	Male	Widow	Junior school	8 months	Good
Patient	59	Female	Widow	Bachelor's degree	1 years and 4 months	Poor
Patient	35	Male	Single	High school diplomas	9 months	Poor
Patient	42	Male	Single	High school diplomas	7 months	Poor
Patient	37	Female	Single	Bachelor's degree	1 years and 5 months	Moderate
Patient	74	Male	Widow	Junior school	1 years	Poor
Patient	56	Female	Widow	High school diplomas	1 years and 6 months	Poor

Data were collected through semi-structured individual interviews, observation, and field notes. In most cases, the first question posed to initiate the interview was “Please describe a day in your life after experiencing hip joint fracture”, and “Describe your living conditions and feelings after experiencing hip joint fracture”. Then, based on the interview guide and participants’ responses, other questions were asked, such as “Has your life changed after the fracture compared to before?”, “How are your personal, social, and occupational relationships after the fracture? Have they changed? Have you felt lonely during this time? What has caused this feeling of yours?”, “What were your most significant concerns after the fracture? Do you consider these concerns resolvable?”, and “What is your current perception about your family and social status and position?”. Additionally, exploratory questions such as “Can you provide more details?” were used as needed during the interviews.

Also, with the progress of the study and the start of data analysis, categories were created that determined the path of subsequent interviews. Subsequent interviews were guided based on the developed theory and the researcher directed new questions based on the prominent and important categories developed. In other words, with the progress of the study, the degree of structuring of the interviews increased and the questions focused on enriching the emerging classes. Questions such as, can you give an example for this condition (disruption of social life)? What did you think when it happened? What was your understanding of life with your limited mobility? What solution did you have in dealing with your new situation? How did you feel after realizing the inability to walk and move like before? What do you do when you feel like this? What are you doing to change your circumstances? Then, based on the responses and data that appeared from the participant, the interviews continued with clarifying and deepening questions.

The duration of the interviews ranged from 45 to 60 min depending on the patients’ mood, willingness, and physical conditions. In some cases, the interviews were carried out in two sessions with an interval from the patient’s rest. The interviews were recorded with participants’ consent using a special recorder, and then transcribed verbatim at the earliest opportunity.

### ***Data analysis***

In this study, the coding steps and paradigm of Corbin and Strauss^21^ were used for the data analysis. According to Corbin’s approach, data analysis consists of several steps, including data analysis to identify the concepts, dimensions, and features of the data (conceptualization), analysis of the findings for context, relating the process to the data analysis (strategies and outcomes), and finally, the integration of the categories and presentation of a theory^21^.

After transcribing the interviews, the data were entered into MAXQDA10 software for data management and organization. The data collection and analysis were performed simultaneously. The analysis was initially very detailed, because all possible scenarios were explored before arriving at any interpretations. The later analyses were more general to develop and validate the interpretations. Grounded theory analysis tools were used to identify the data related to the context, process, and outcome. Asking questions about the data and conducting comparisons (continuous and theoretical comparison) were the main tools and the focus of this analysis. Other analysis strategies used included thinking about the different meanings of a word, paying attention to body language, paying attention to the emotions expressed and the situations that have incited the emotions, drawing on personal experiences, searching from the perspective of temporal words, thinking about metaphorical words, and searching for negative (opposing) cases (69, 136).

### Trustworthiness

Corbin and Strauss do not consider quality and validity synonymous, as they believe that high-quality findings have creative, thoughtful, and unique components, and they do not use the terms ‘validity’ and ‘reliability’, because these words carry too much quantitative weight. They prefer to use the term ‘rigor, as it indicates that the findings are trustworthy and credible and reflect the experiences of the participants, researchers, and readers about the phenomenon under study. Therefore, ten criteria, including fit, applicability of the findings, concepts, contextuality, logicality, depth, variability and possessing a spectrum, creativity, sensitivity, and evidence of reminders, were used to assess the study’s quality and increase its rigor.

## Results

**Figure 1:**
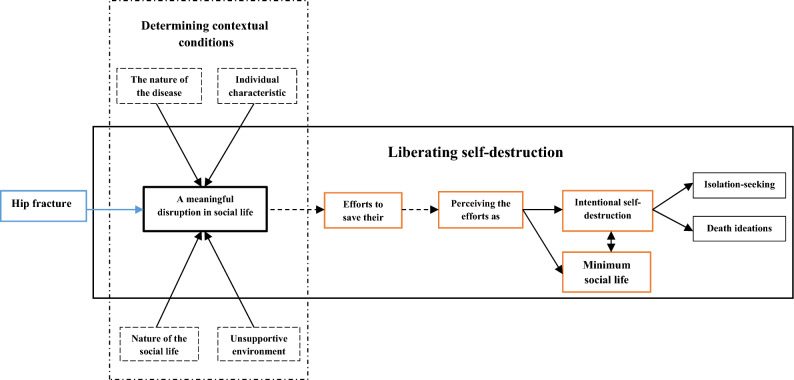
Model of the process of social death.

Generally, the analysis of the research data to explain the process of social death formation (Fig. 1) in individuals with hip joint fracture led to the development of a core category, 16 main categories, and 55 subcategories. These categories and subcategories resulted from the classification of 1712 primary codes and 212 primary concepts. The core category of the study was liberating self-destruction. Table 2 presents the concepts, categories, subcategories as well as the core category.

**Table 2:** Core category, category and subcategory of study.

		Category	Subcategory
Core category “Liberating self-destruction”	Contextual conditions	Determining contextual conditions	The nature of the disease Individual characteristics Unsupportiveenvironment Nature of the social life
	Action/interactional strategies The process of social death	A meaningful disruption in social life	Personal problems after a fracture The social consequences of losing physical mobility Consequences of losing roles Inability to manage social life issues Social identity in danger Unfulfilled social demands
		Efforts to save their life	Resorting to compensatory measures Kindness behaviors for more interaction Appeal to spiritual healing Reducing expectations and giving in to the minimum Trying to do daily activities with physical limitations after fracture Demand for better medical conditions Ask for a listening ear
		Perceiving the efforts as futile	Understanding the deterioration of physical condition and disability Thoughts on the possibility of failure of therapeutic efforts The mentality of lagging behind the flow of life (a person's perception of lagging) Remembering unfortunate experiences
		Minimum social life	Practical matching of expectations with physical abilities Efforts to maintain social function
		Intentional self-destruction	Rejecting support Ignoring concerns for others Intensified self-suffering Destroying social identity Effortlessness, and Detachment
	Outcomes	Isolation-seeking	Disturbed interactions Constantly avoiding social activity
		Death ideations	Getting fed up with continuing to live a miserable life Preferring death to life in purgatory Death thoughts

The main objective of the coding process was conceptualization and data classification. Therefore, all possible findings and meanings in the data were identified and a conceptual label was assigned to the data. In this study, the main categories included determining contextual conditions, gradual disintegration of meaningful social life, attempts to save life, understanding the futility of attempts, minimal social life, intentional self-destruction, isolation-seeking, and death ideations.

### Contextual conditions

The context influencing the process of social death formation, labeled as ‘determining contextual conditions’, was identified with four main categories, including the nature of the disease, individual characteristics, unsupportive environment, and nature of the social life (Table 2).

### Nature of the disease

The nature of the disease (characteristics of the disease and treatment) plays an important role in the patient’s response to it. This subcategory can include the many surgeries, the difficulty of healing, the problem of caregivers who, even during hospitalization, do not talk to the patient, etc.

Some specific characteristics of hip joint fractures facilitate the basic process of social death formation. Therefore, individuals with hip joint fracture show different reactions commensurate with their experience of the various problems caused by the fracture. According to the analysis of the interviews conducted with the patients, the most important characteristics of this type of fracture that have led to greater struggles for them were divided into four subcategories: incurable, progressive, sudden, and chronic and non-pathological nature.

Most of the patients and caregivers believed that their physical disability and problems had worsened over time to the extent that they had not anticipated these conditions. One of the patients who had visited the clinic after a year stated:

*If my treatment went well, I would not have come here again after a year. I have had multiple surgeries. The second time, my surgery site got infected, and those were the most painful days of my life. The discharge from the surgical site smelled terrible and was abundant, but thankfully, I recovered after a month and was discharged again, but I have not been able to walk anymore.*

### Individual characteristics

Individuals with hip joint fracture have shown different reactions to the fracture crisis based on their personality and characteristics. The most important individual characteristics were presented in six subcategories: belief in their own passivity (personal relationships), having been socially useful in former times (social relationships), attachment avoidance, deep belief, compassion avoidance, and lack of gratitude towards others.

The perception and belief that individuals have about themselves can be a facilitator or barrier of the formation of social death. These characteristics have a direct impact on individuals’ actions, reactions, and emotions. Patients who perceived themselves as more passive than in the past were more susceptible to social death, because their expectations and abilities had suddenly gotten disrupted. Most of the individuals stated that they had lost their independence and self-management as a result of no longer pursuing their previous life engagements and activities. 

A 34-year-old man who felt satisfied with his independence and activities before his fracture expressed his current conditions by comparing them to the past:

*For someone like me who was constantly active and working all day long and was independent since childhood without getting tired, it’s really hard to suddenly not even be able to stand on my feet, let alone go to work and earn money.*

### Non-supportive therapeutic environment

As for the factors influencing the formation of social death, a non-supportive therapeutic environment has an undeniable role, which includes two subcategories: defective treatment culture and defective post-discharge care culture. The treatment of patients with hip joint fracture in hospitals is a chronic process, and their therapeutic response and recovery depend on various factors. Inadequate training for these patients and the failure to meet their care needs were some of the fundamental problems they faced, which led to harsh consequences such as non-recovery, treatment abandonment, and loss of identity following a sense of humiliation and burden, which can be features of the basic process of social death.

The defective treatment culture had various characteristics from the patients’ perspective, including the primary concepts, the healthcare personnel’s neglect of training, discriminatory views in treatment, lack of attention to holistic care, dissatisfaction with the treatment services, patients’ perceived unimportance in their own treatment process, and the different conditions prevailing in different treatment environments. Since hip joint fracture has a lengthy and chronic treatment process, post-discharge care and at-home care also play a fundamental role in the recovery process of these patients. The interviews showed that the care culture in hospitals and at home was defective and inadequate. Additionally, the analysis of the interviews also revealed that emotional, psychological, and social support has been less addressed among these patients. In this regard, one of the patients stated:

*Here, they treat you like a lifeless object; they give you an injection and leave. It’s like we don’t have a life or any worries. Or we like to sleep on the bed. They don’t talk to us; they don’t explain what we need to do to maybe get better sooner.*

Another important influential factor in the process of social death in individuals with hip joint fracture was the main subcategory called non-supportive family conditions, which included primary concepts, a disturbed and unsafe family (disturbed with the patient’s fracture), non-supportive family, being rejected by family members, and weakened foundation of family life (divorce, separation, etc.).

### Nature of the socioeconomic life

The nature of the patients’ social life is closely linked to the pre-fracture work, family and social conditions; however, all these individuals have faced disorders in the nature of their social life that can facilitate the formation of social death. Based on the conducted interviews, the nature of the socioeconomic life is made up of two sub-categories: the characteristics of the surrounding social community (colleagues, neighbors, and relatives) and economic-welfare conditions.

In this study, most patients complained about the rejecting society (friends, neighbors, and relatives), social discrimination against the patient, and the lack of understanding for the patient’s mobility situation by the surrounding community; this issue is to the extent that they have lost their position in their work environment and community with the intensification of their disabilities. In addition to no longer having the status of an active member of the society due to the prevailing norms, they have been subjected to various social prejudices in relation to their new physical and mental conditions and have been rejected overall.

One of the patients stated that after long-term non-recovery, the people around them (neighbors and relatives) accused them of various diseases and expelled them:

*People in the neighborhood and my relatives say that after my hospitalization I have gone mad and don’t talk to anyone and talk to myself, and say a lot of other things that hurt my feelings. Now they treat me as if I have a strange disease and they avoid me. They no longer come to my place like they used to.*

Economic-welfare conditions are one of the important factors contributing to the quality of social life, and individuals with better economic status can have easier access to medical and care services. Nevertheless, in most of the conducted interviews, financial concerns were among the prominent problems of these patients. A limited number of patients had a high economic status.

### Action/interactional strategies

The continuous comparative analysis of the data indicated that the main concern and worry of most participants after a period of time following hip joint fracture was “maintaining a meaningful social life” after perceiving “a meaningful disruption in social life” and the inability to return to their previous social life. To face the challenges of hip joint fracture in their now-disrupted life due to contextual and intervening factors, the patients used the strategy of “making efforts to save their life”. In some patients, despite “perceiving the efforts as futile,” the range of effective and appropriate strategies as “efforts to save their life” results in achieving a “minimum social life”; however, this stage of the patients’ life is entirely fragile and may be affected by various factors, especially unsuccessful treatment and the nature of the fracture, leading to intentional self-destruction by a defective loop (Table 2).

The second category of strategies used by patients as the basic process of social death formation was called “intentional self-destruction.” “Intentional self-destruction” includes suppressive strategies in various physical, mental, psychological, and social dimensions, such as “rejecting support, ignoring concerns for others, intensified self-suffering, destroying social identity, effortlessness, and detachment”, which most patients use at some point during the long process of hip joint fracture treatment to achieve relief and escape their painful situation, leading to outcomes such as “isolation-seeking” and ultimately “death ideations” (Fig. 1).

### Rejecting support

After some time from the initial fracture and upon observing others’ behavior, most patients avoided and rejected the support of their families and acquaintances and complained about their useless recommendations and control.

An older woman aged 79 years old said,

* “I don’t want to cause any trouble for my family. I don’t know when I will get better, and I have become a burden for them. Only one of my daughters takes care of me, and I don’t want to bother her anymore. I prefer that they hire a caregiver for me.*

### Ignoring concerns and responsibilities towards others

After a while, most patients reached a stage where they became indifferent and unconcerned towards all the events around them and their acquaintances, considering their family, friends, and colleagues as well as their family-related and occupational issues unimportant and becoming totally indifferent.

They also avoided any kind of social contact and engaged in no social activities, preferring a passive and isolated lifestyle, which is a primary and important characteristic of the basic process of intentional self-destruction.

A male participant who preferred being alone said,

*I don’t have the patience for anyone. They all give me useless advice and insist on things. I like to be alone. If I didn’t need help going to the bathroom and doing my chores, I wouldn’t see anyone. I don’t have the will to communicate and talk to people anymore; I don’t have the motivation. No one is important to me now.*

### Destruction of the social identity

Destruction of the social identity is one of the main strategies used by patients for self-destruction. By destroying their social identity, the patients aim to reduce their own and others’ expectations. Moreover, the perception of worthlessness in the eyes of others often exacerbated after losing their job and facing difficulty in fulfilling their social roles. Regarding the feeling of futility and inability to improve their conditions, one patient said:

*This kind of life is difficult for me –who used to be so active. I feel that I don’t play any role in my own life or my family’s life, and these thoughts make me feel futile. I no longer recognize myself and I’m not who I used to be and I can’t do anything for myself because I am incapable of it.*

### Intensified self-suffering (psychological aspect)

One of the main strategies for the formation of social death in the patients was their psychological conflicts, which, given their predisposing context, most of the patients had experienced in the form of self-suffering and aggravation of their psychological distress at some stage of the hip joint fracture treatment process. With intensified depression and other mental health problems and feeling worthless, the patients reduce any expectations of themselves and gradually move towards social withdrawal and isolation.

Most patients had experienced a range of suffering following hip joint fracture. The collection of sufferings was made up of primary concepts such as feelings of depression, apathy, irritability, excessive grief and sorrow, severe longing, chronic pain and suffering, and a decline in the patient’s mood. One patient said:

*I am constantly tormented and struggling with myself, and now my mind is so preoccupied with this problem that I am depressed. I hate being in this condition.*

### Effortlessness and detachment

Effortlessness and detachment are the main characteristics of the process of social death formation. This category is procedural, and patients gradually become physically and mentally exhausted due to the drivers of treatment withdrawal. With increasing avoidance and indifference towards social interactions, patients eventually come to the realization of being detached. Often, as more time elapses from the initial fracture, patients become tired of the treatment process and get disillusioned with ever recovering due to their repeated failures and multiple complications. Some patients even avoid hospitalization and refuse continuing treatment. Most patients do not adhere to the recommended rehabilitation programs after getting discharged from the hospital. These factors contribute to treatment discontinuation in these individuals.

One patient explained about his exhaustion after a year and a half from the hip fracture,

*I am so tired that I no longer think about anything; I mean, I don’t want to get well anymore. I just want to be able to do simple things for myself until I die. I rather die but don’t go back to the hospital for another surgery.*

The stage at which the patients physically surrendered during the process of treatment of fracture is decided by the determining contextual factors, including personal characteristics, the environment, unsupportive family, and especially the nature of their social life.

### Consequences

**Figure 2:**
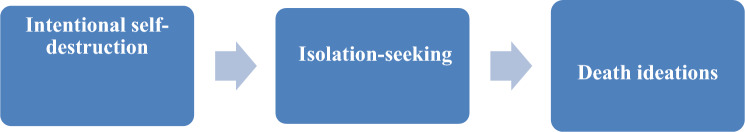
Dimensions and characteristics of the consequences and process of Intentional self-destruction (the process of social death).

Most of the strategies employed by the patients in the basic process of social death formation ultimately led to their isolation and suicidal ideation (Fig. 2). Throughout their treatment process, all of these patients experienced a forced or voluntary reduction in social communications and activities. One patient expressed having become indifferent towards social relationships and participation in various gatherings after feeling that their social identity had been destroyed and said:

*After this incident, I lost my identity and status in others’ eyes. I feel like my identity has become nothing to my friends and family. That’s why I have distanced myself from everyone.*

What was evident in most interviews was that many patients had reached a state of social withdrawal and suicidal ideation as a result of feeling exhausted and desperate, and they expressed readiness and preference for death. In other words, social withdrawal can be the beginning of suicidal ideations. With their sense of despair, some patients described their life as torturous and limbo-like and expressed that with such hardship, they preferred a purposeless life devoid of all activities until their death. They were kind of ready to die:

*The reality is that I don’t know when it is going to get better fully; I’ve been waiting for too long and now I’m just stuck in my daily life, feeling tired and hopeless. That’s why I prefer to do nothing and go nowhere to not make myself feel even more miserable. I feel like a dead man walking who only eats and does nothing else.*

Given their background and social context, some patients experienced suicidal ideations during the long process of their treatment, and they reached a certain acceptance and readiness for dying regardless of their religious beliefs. In other words, suicidal ideations had overshadowed the continuation of life. Younger individuals had suicidal thoughts, while older individuals believed in the inevitability of death based on their religious beliefs and expressed no fear of it. Despite the different dimensions of suicidal ideation, the goal was mostly to relieve themselves and their families. Some patients also believed that their families were ready for their death in addition to themselves.

By stating the futility of therapeutic counseling, a patient spoke of their suicidal thoughts as follows:

*I think a lot about suicide and getting rid of this situation. I know even if I have surgery a hundred times and suffer, I won’t get better in the end. I might end up crippled, maybe limp when walking, and I’ll get twenty years older. I can’t tolerate this kind of improvement. What’s the point?*

### Core category

“Liberating self-destruction” as a core category was closely tied to the contextual factors and the strategies employed by the patients and their consequences, since the ultimate goal of the patients was “to be freed from a disrupted social life” after hip joint fracture and understanding the futility of any efforts to save their social life (Fig. 1).

## Discussion

As an acute trauma following a fall or accident, hip joint fracture has a specific treatment in the form of surgery^22^. Nevertheless, the life story of patients with different conditions showed that dealing with and treating this type of fracture is a complex and unique experience in different individuals with distinct personal and social characteristics due to its chronic and unpredictable nature^10^.

The process of social death formation for patients with hip fracture is a dynamic, multidimensional, and evolutionary process that may begin with understanding the failure of their initial efforts to save their social life and achieve a minimal form of it at least. Evidently, some individuals do not make any initial efforts to achieve a minimal social life and immediately employ intentional self-destructive and repressive strategies after learning that their meaningful social life has been disrupted, which is in direct relation to their goal of freeing themselves from their new conditions.

In the present study, with the gradual deterioration of their meaningful social life and understanding the loss of their social identity, patients had resorted to intentionally self-destructive strategies to liberate themselves from their difficult social conditions that could ultimately lead to isolation and suicidal ideations. Studies have also pointed out the threat to the social identity of these patients and the need to pay attention to the social aspect of their life^2^. Limited studies have been conducted on the nature and characteristics of social death in different patients^9,17,23^. Jan Králová highlighted the susceptibility of patients with difficult chronic conditions and lengthy treatment processes to experiencing social death before their physical death^17^. found that the difficult recovery and disrupted social life due to restricted mobility following hip fracture had made patients become socially isolated, to the extent that their ability to return to their normal social life and former state of health had been negatively impacted^4,24,25^.

In the present study, patients who had developed a deliberate and conscious self-destruction to free themselves from their difficult social conditions after hip joint fracture experienced social death. Social death is manifested when people with disabilities live in a society that rejects them and are considered unacceptable for social participation and continuing their social life, and while they are alive, they appear dead from their own or others’ view. Some groups are more susceptible to these perceptions in the society, and various patients, especially those with difficult and lengthy treatment processes, are among them^15,26^. The concept of “social death” has also been used by some medical researchers to mean the severe endangerment of “physical, mental, and social health”^17^. Some chronic and wearying physical illnesses are more prone to weakening the patient’s social identity and social interactions and causing their abandonment by others^10^. Social death, i.e., the extinction and end of social identity and social life, shows that death and dying are not only a physical phenomenon or even a psychological loss, but a form of liberation and social loss^14^.

One of the findings revealed the concerns of the participants, particularly in the early stages of their fracture, regarding their inability to perform their previous roles and responsibilities. While individuals’ reactions to ongoing disabilities varied, many patients employed denial and avoidance strategies, ultimately seeking neglect of their previous concerns and responsibilities by ignoring their own conditions and of those around them after losing their job and seeking isolation from their family, colleagues, and friends. In other words, they considered themselves irresponsible and regarded events as insignificant in order to release themselves from any concerns about themselves or others, but ultimately, their self-suffering and social isolation intensified instead of gaining more adaptation. Numerous studies have also noted that patients with hip joint fractures become unable to continue some of their previous social relationships and roles^27–29^.

The results also showed that patients gradually became unmotivated to participate in any social activities as a result of voluntary or compulsive loneliness. Keyes’s theory also suggests that social participation and vitality are important dimensions of social health. Naturally, individuals whose physical health has been compromised have a reduced participation in activities and social relationships inevitably^30,31^. Hence, patients’ social activities decrease during their lengthy treatment processes due to the physical and mental conflicts they experience^13^.

Social death is, in a sense, the death of vital social relationships. Social vitality exists through contemporary relationships and creates connections between backgrounds and identities that give meaning and shape to human life^9,10,26^. Some of these relationships are with relatives, friends, and colleagues. When patients become indifferent toward their relationships with others, they naturally lose their social vitality. Moreover, when social vitality is lost, individuals will not have the motivation to collaborate with essential social, economic, political, religious, and educational institutions and will become socially isolated^23,32^.

In the present study, some patients had reached a state of identity loss as a result of their perceived loss of independence. One of their most important coping strategies was intentional self-destruction for justifying their withdrawal and abandonment of any attempts for social participation and social life. Sociological studies also suggest that when a particular group of patients are marginalized, isolated and rejected, a social action is taken against them, resulting in a sense of identity loss and reactions such as anger and violence or gradual withdrawal and silence for self-liberation^32,33^.The intensification of the conditions and the rejection of the patients also reduce their ability to rejoin and return to the main community. The more severe is this social rejection, the less likely are the group of patients able to reintegrate into the main community, resulting in their being in conflict with the community and escaping it altogether^31,33,34^. Another qualitative studies emphasized the need for maintaining independence and responsibility for having and continuing a meaningful social life in patients with hip fracture^22,35^.

In a qualitative study by Olsson et al., patients who were dissatisfied with their appearance and turmoil had accepted that they were unable to continue their life and that they had lost everything by surrendering to depression and having no motivations. They also perceived that problems resulting from hip joint fracture were inevitable due to their weakness and felt increasingly defeated. They believed that they had lost their welfare, self-confidence and the physical health and resilience necessary for coping and adaptation, and by considering themselves guilty, were trying to accept their limitations^22^. This finding is in line with the basic process of intentional self-destruction in the present study.

In the process of social death formation, some individuals had come to suicidal ideations based on their different circumstances, context, and self-destructive strategies. Suicidal ideation is a very dangerous consequence that often arises when patients become tired of their life when perceiving the agonizing nature of life, becoming disenchanted with their purposeless existence, and losing their desire for living. With such repressive perceptions, individuals often prefer death over life at this stage^36^. In a study by Haleh et al., patients who had suffered from facial burns preferred death over living with their disability due to their perceived lack of social status^37^.

## Conclusion

In response to the research question of how social death is formed in individuals with hip joint fracture, the study findings suggest that the process of social death formation is dynamic, multidimensional, evolutionary, and progressive in these patients and is dependent on contextual conditions. Patients experience different degrees and stages of this process based on their own conditions and situation. Patients with hip joint fracture and restricted mobility face disruptions in their meaningful social life under the influence of factors such as the nature of the disease, their personal characteristics, unsupportive environment, and their socio-economic life. Affected by these factors, the patients used two categories of strategies and tactics in coping with their disrupted life, ranging from effective (positive) to ineffective (self-destructive). Effective strategies emerged as attempts to save their life and led to at least a minimal social life for these individuals, who maintained some level of social functioning by accepting minimal standards. Nevertheless, these conditions are highly fragile and may even be exacerbated by worsening conditions and contextual factors, leading to further disruptions in their social life. Moreover, some other patients employed ineffective strategies known as intentional self-destruction when faced with their disrupted social life, which was considered the basic process of social death formation in these patients that ultimately led to consequences such as isolation and suicidal ideations.

### Limitations

One of the limitations of qualitative studies is the dependence of the research findings on various conditions, particularly time and location^38^, and this study is no exception to this rule.
